# The Rho-associated kinase inhibitor fasudil can replace Y-27632 for use in human pluripotent stem cell research

**DOI:** 10.1371/journal.pone.0233057

**Published:** 2020-05-12

**Authors:** Seongjun So, Yeonmi Lee, Jiwan Choi, Seoon Kang, Ji-Yoon Lee, Julie Hwang, Joosung Shin, James R. Dutton, Eul-Ju Seo, Beom Hee Lee, Chong Jai Kim, Shoukhrat Mitalipov, Soo Jin Oh, Eunju Kang

**Affiliations:** 1 Stem Cell Center, Asan Institute for Life Sciences, Asan Medical Center, University of Ulsan College of Medicine, Seoul, Republic of Korea; 2 Department of Convergence Medicine, Asan Institute for Life Sciences, Asan Medical Center, University of Ulsan College of Medicine, Seoul, Republic of Korea; 3 Stem Cell Institute, University of Minnesota, Minneapolis, Minnesota, United States of America; 4 Medical Genetics Center, Asan Institute for Life Sciences, Asan Medical Center, University of Ulsan College of Medicine, Seoul, Republic of Korea; 5 Department of Pathology, Asan Institute for Life Sciences, Asan Medical Center, University of Ulsan College of Medicine, Seoul, Republic of Korea; 6 Center for Embryonic Cell and Gene Therapy, Oregon Health & Science University, Portland, Oregon, United States of America; Purdue University, UNITED STATES

## Abstract

Poor survival of human pluripotent stem cells (hPSCs) following freezing, thawing, or passaging hinders the maintenance and differentiation of stem cells. Rho-associated kinases (ROCKs) play a crucial role in hPSC survival. To date, a typical ROCK inhibitor, Y-27632, has been the primary agent used in hPSC research. Here, we report that another ROCK inhibitor, fasudil, can be used as an alternative and is cheaper than Y-27632. It increased hPSC growth following thawing and passaging, like Y-27632, and did not affect pluripotency, differentiation ability, and chromosome integrity. Furthermore, fasudil promoted retinal pigment epithelium (RPE) differentiation and the survival of neural crest cells (NCCs) during differentiation. It was also useful for single-cell passaging of hPSCs and during aggregation. These findings suggest that fasudil can replace Y-27632 for use in stem research.

## Introduction

Human pluripotent stem cells (hPSCs), such as embryonic stem cells (hESCs) and induced pluripotent stem cells (hiPSCs), are valuable research tools in the fields of developmental biology, disease modeling, drug screening, and regenerative medicine [1–3]. However, hPSCs are difficult to culture because of their poor survival, especially when they are detached from surfaces or dissociated for freezing [4–6]. Hence hPSCs were initially cultured on feeder layers to increase their survival and maintain pluripotency.

Mouse embryonic fibroblasts (MEFs) are the most commonly used feeder cells in research but may have limited efficacy with human cells due to xenogeneic contamination [6, 7]. Another disadvantage of MEFs is that the use of any feeder layer itself leads to batch- or lab-dependent variation; additionally, feeder layers increase workload, which subsequently limits large-scale hPSC culture [8, 9]. To overcome these problems, additional methods have been introduced, such as culturing hPSCs in suspension with microcarriers or on synthetic polymers [10]. As a result, feeder-free hPSC cultures became possible when hPSCs were grown on an extracellular matrix with specialized small molecules and growth factors [10, 11].

In 2007, Y-27632 ([1R,4r]-4-[(R)-1-aminoethyl]-N-[pyridin-4-yl] cyclohexane carboxamide), the first small molecule to inhibit the Rho-associated kinase (ROCK) pathway, was reported to increase the survival and growth of PSCs; since then, it has been used extensively in stem cell research [12]. It has also been used to encourage PSC differentiation in endodermal lineage cells [13] and insulin-producing cells [14], and for promoting the maturation or maintenance of differentiated cells [15–17]. The ROCK signal activates the phospho-myosin light chain (pMLC), which contracts intracellular actomyosin and is known to induce apoptosis in dissociated cells [18].

Recently, many researchers have used ROCK inhibitors as essential small molecules for PSC culture. Since Y-27632 was developed and put into use, researchers have primarily resorted to this ROCK inhibitor for PSCs. However, the synthesis of Y-27632 consists of seven steps, and the yield is only 45%, which tends to increase its cost [19]. There are several ROCK inhibitors available on the market, including RKI-1447, GSK429286A, H-1152, SLx-2119, TC-S 7001, and fasudil. In particular, fasudil (HA-1077, 5-[1,4-diazepan-1-ylsulfonyl] isoquinoline) is approved for clinical use and is already used in Japan and China for the prevention of cerebral vasospasm after subarachnoid hemorrhage [20]. Also, it is synthesized in one step and has a higher yield than Y-27632 (73.1% vs. 45% in Y-27632) [21].

In the present study, we compared the effectiveness of fasudil and Y-27632 during long-term xeno-free growth and maintenance of hPSCs in conditions of freezing, thawing, and splitting. Fasudil proved to be useful for inducing 3D aggregation of PSCs and for their differentiation to retinal pigmented epithelial (RPE) cells, and neural crest cells (NCCs) (Table 1).

**Table 1:** List of hPSC lines used.

Cell line	hES1	hES2	hES3	hiPS1	hiPS2
Origin	Blastocysts			40-yr, Fibroblasts	49-yr, Fibroblasts
Pluripotency gene expression	√	√	√	√	√
Germ layer differentiation gene expression	√	√	√	√	√
Pluripotency protein expression	√	√	√	√	√
Karyotyping	√	√	√	√	√
Teratoma assay	√	√	√	√	√
Cell growth	√	√	√	√	√
Freezing and thawing		√		√	
Live and apoptotic cell analysis			√		√
Cell proliferation analysis			√		√
RPE cell differentiation				√	
NCC differentiation				√	
Single-cell passaging			√		
3D aggregation			√		

Three hES (hES1, hES2, and hES3) and two hiPS (hiPS1 and hiPS2) cell lines were used in this study. All the lines were characterized by qPCR, karyotyping, and teratoma assay. Each experiment was performed with randomly selected hPSC lines. hPSC: human pluripotent stem cell, hES: human embryonic stem cell, hiPS: human induced pluripotent stem cell, RPE: retinal pigment epithelium, NCC: neural crest cell

## Materials and methods

### Ethics and governance

Informed consent was obtained from participants for the use of human samples and use was approved by the institutional review board (IRB: 2017–0260 and 2019–0117) of Asan Medical Center. Experiments on live vertebrates were performed in compliance with the regulations of the Asan Institute for Life Sciences and were approved by the Asan Institutional Animal Care and Use Committee (IACUC: 2018-12-269). All experiments were performed according to relevant guidelines and regulations.

### Analysis of small molecule stability

HPLC was performed as previously described [22]. Briefly, samples (50 uL) were precipitated with 150 μL of cold acetonitrile containing carbamazepine as internal standard (CBZ, 10 ng/ml) and agitated with a vortex mixer before centrifugation at 3400 rpm and 4°C for 20 minutes. The supernatants were then analyzed by liquid chromatography-electrospray ionization tandem mass spectrometry (LC-ESI/MS/MS) with an Agilent 1200 series HPLC system (Agilent Technologies, Wilmington, DE, USA) and API 4000 LC-MS/MS system equipped with a Turbo V Ion-Spray source (Applied Biosystems, Foster City, CA, USA) operated in the positive ion mode. Chromatographic separation was carried out on a Hypersil GOLD C18 column (50 × 4.6 mm i.d., 5 μm, Thermo Fisher Scientific, Waltham, MA, USA) for Y-27632, and on an Atlantis dC18 column (50 × 2.1 mm i.d., 3 μm, Waters, Milford, MA, USA) with a Security Guard C18 guard column (2.0 × 4.0 mm i.d., Phenomenex, Torrance, CA, USA) for fasudil. Detection involved multiple reaction monitoring (MPM) of the transitions of m/z 292>99 for fasudil, m/z 248>95 for Y-27632 and m/z 237>194 for CBZ (international standard). The retention times of fasudil and Y-27632 were 2.92 and 1.94 minutes, respectively. Acquisition and analysis of data were performed using Analyst software (ver. 1.5.2, Applied Biosystems). For all LC-MS/MS analyses, the peak areas of fasudil and Y-27632 were expressed relative to the internal standard (CBZ) peak area for each trial.

### Cell culture

Three hESC lines and four hiPSC lines were maintained, based on the hPSC research guidelines of the Stem Cell Center, Asan Medical Center (Seoul, Republic of Korea). The hPSCs were cultured on vitronectin-coated (5 μg/ml; Life Technologies, Grand Island, NY, USA) culture plates in Essential 8 medium (Life Technologies) at 37°C, 5% CO_2_. The cultures were passaged every week in clusters by chemically dissociating the PSCs using lab-made reagent (0.4% sodium citrate, S4641, Sigma-Aldrich, St. Louis, MO, USA) or commercial reagent (Gentle Cell Dissociation Reagent, Stem Cell Technologies, Vancouver, BC, Canada). Dissociation using lab-made reagent was carried out as described previously [23], and for comparison with lab-made reagent, the commercial reagent was used according to the manufacturer’s instructions. The hPSC clusters were transferred onto prepared vitronectin-coated culture plates supplemented with fasudil (10 μM; Adooq, Irvine, CA, USA) or Y-27632 (10 μM; Sigma-Aldrich).

### Freezing and thawing

Before freezing hPSCs, 10 μM of ROCK inhibitor (Y-27632 or fasudil) was added to the culture medium for 30 minutes. hPSCs were collected in small clumps with lab-made reagent, centrifuged (300 g for 1 minute), and resuspended in Essential 8 medium. Cell suspensions were mixed slowly with freezing medium (20% DMSO in culture medium) in a final volume ratio of 1:1and dispensed into cryovials (Life Technologies), which were immediately placed into CoolCell LX Freezing Containers (Sigma-Aldrich). The vials were stored overnight in a -80°C freezer that provides a cooling rate of approximately 1°C/minute. The following day, the vials were placed for storage either in a liquid nitrogen tank or at -80°C. For thawing, they were rapidly warmed in a 37°C water bath for approximately 1 minute until the ice disappeared. The cell suspensions were then transferred to 15 ml centrifuge tubes and slowly mixed with 5 ml of warm culture medium. After centrifugation (300 g for 1 minute), the supernatant solutions were removed, and cell pellets were resuspended with 1 ml of fresh culture medium. The cells were then plated in vitronectin-coated plates and cultured in the conditions described above.

### Alkaline phosphatase activity

An Alkaline Phosphatase Substrate Kit I (System Biosciences, Palo Alto, CA, USA) was used to analyze alkaline phosphatase activity within clusters.

### Cell counting

To examine cell growth, sub-confluent cells were dissociated into clumps using lab-made reagent (sodium citrate) or commercial reagent. The cell clumps were dissociated into single cells with 0.25% trypsin-EDTA for single-cell counting. These cells were not used for further culture.

### Living and apoptotic cells

Apoptosis analysis was carried out using a Dead Cell Apoptosis Kit with Annexin V Alexa Fluor 488 & Propidium Iodide (Invitrogen). After staining with Annexin V/PI, floating cells were collected and retained and the attached, Annexin V/PI-stained cells were further counted using a FACS (Canto II, BD Biosciences).

### Cell proliferation analysis

To determine proliferation, hPSCs were collected and fixed with 10% neutral buffered formalin (BBC Biochemical, Mount Vernon, WA) for 30 minutes at room temperature on the third day after the split. Fixed cells were washed twice with phosphate‐buffered saline (PBS, Hyclone, Chicago, IL, USA) and permeabilized with 0.05% Triton X-100 in 0.01 M sodium citrate for 30 minutes at room temperature. Ki-67 (Abcam, Cambridge, UK) primary antibody was diluted at 1:200 in PBS containing 10% fetal bovine serum (FBS, Life Technologies) and refrigerated overnight. Thereafter the cells were washed three times with PBS and incubated for 1 hour in the refrigerator with Goat Anti-Rabbit IgG H&L (1:500, Alexa Fluor 555, Abcam) and counted using a FACS (Canto II, BD Biosciences). The FACS data were analyzed with FlowJo xV.0.7 software (Tree Star, Ashland, OR).

### Quantitative polymerase chain reaction (qPCR)

Total RNAs of hPSCs cultured in the presence of fasudil or Y-27632 were isolated using RNeasy Mini Kits (Qiagen, Valencia, CA, USA), and reverse transcription was performed with cDNA synthesis kits (PCR Biosystems, London, UK). The results were confirmed using a conventional real-time polymerase chain reaction (PCR) analysis. Quantitative reverse transcriptase PCR (RT-qPCR) was then performed using the relevant gene-specific primers, Power SYBR Green PCR Master Mix, and a QuantStudio 6 Flex Real-Time PCR System (Applied Biosystems, Foster City, CA, USA).

The primers used were: *OCT4* F-5′-GACAGGGGGAGGGGAGGAGCTAGG-3′, R-5′-CTTCCCTCCAACCAGTTGCCCCAAA-3′, *SOX2* F-5′-AGCTACAGCATGATGCAGGA -3′, R-5′-GGTCATGGAGTTGTACTGCA-3′, *NANOG* F-5′-TGAACCTCAGCTACAAACAG-3′, R-5′-TGGTGGTAGGAAGAGTAAAG-3′, *AFP* F-5′- AGGGAGCGGCTGACATTATT-3′, R-5′- CAGAGAATGCAGGAGGGACA -3′ [24], *TBXT* F-5′-ACCCAGTTCATAGCGGTGAC-3′, R-5′- CCATTGGGAGTACCCAGGTT -3′ [25], *PAX6* F-5′- TGTGTGCTCTGAAGGTCAGG-3′, R-5′- CTGGAGCTCTGTTTGGAAGG-3′ [26]. The qPCR was performed in a final volume of 20 μl containing 10 μl SYBR Green master mix, 2 μl cDNA (50 ng/μl), 0.5 μl each forward and reverse primer (10 pmol) and 7 μl nuclease-free water, and the annealing temperature was 62~65°C. The PCR data were analyzed and normalized with *GAPDH* expression, using QuantStudio 6 and 7 Flex software (Applied Biosystems).

### Immunocytochemistry

Fasudil-treated hPSCs were seeded onto vitronectin-coated plates. Five days after plating, hPSCs were fixed in 10% neutral buffered formalin overnight in a refrigerator (2–8°C), washed twice with PBS, and permeabilized with 0.05% Triton X-100 in 0.01 M sodium citrate for 30 minutes at room temperature if required. Primary antibodies were diluted from 1:100 to 1:500 in PBS containing 10% FBS and incubated overnight in the refrigerator. The primary antibodies, TRA-1-60, SSEA4, and Oct4 (1:100, Stemgent, Cambridge, MA, USA), were used for pluripotency analysis, and PAX3, PAX6, SOX10 and MITF antibodies (1:200, Abcam) were used for differentiation analysis. After incubation with primary antibodies, cells were washed three times with PBS and incubated for 1 hour at room temperature with secondary antibodies. The secondary antibodies were Goat Anti-Mouse IgG H&L (1:1000, Alexa Fluor 488, Abcam), Goat Anti-Rabbit IgG H&L (1:500, Alexa Fluor 555, Abcam) and Goat Anti-Rabbit IgG H&L (1:200, Alexa Fluor 647, Abcam). Samples were imaged and captured using a Carl Zeiss inverted microscope with an AxioCam MRc Rev 3 digital camera (Zeiss, Oberkochen, Germany; Confocal Imaging Core Laboratory, ASAN Institute for Life Sciences, Seoul, Republic of Korea). The expression population was counted using a FACS (Canto II, BD Biosciences). The FACS data were analyzed with FlowJo xV.0.7 software (Tree Star, Ashland, OR).

### Teratoma formation

Teratoma formation experiments were performed by injecting hPSCs (maintained in the presence of fasudil for three months and detached from vitronectin-coated plates as cell clumps containing fasudil) into the femoral region of 7-week-old NOD-SCID Gamma mice (NSG, The Jackson Laboratory, Bar Harbor, ME) using a 1 ml syringe (Korea Vaccine Co., Seoul, Republic of Korea). Mice with tumors were euthanized, and teratomas were isolated, sectioned, stained with hematoxylin and eosin (H&E) and histologically characterized for the presence of representative tissues.

### Karyotyping

Karyotyping was carried out on hPSC lines (hES1, hES2, hES3, hiPS1, and hiPS2) maintained in the presence of 10 μM fasudil over three months by standard G banding at the Medical Genetic Center, Asan Medical Center (Seoul, Republic of Korea).

### Differentiation

Retinal pigment epithelium (RPE) cells were generated by *in vitro* differentiation of hPSCs, as previously described [26]. Briefly, hPSCs were plated at 2 × 10^4^/cm^2^ on plates coated with vitronectin in the Essential 8 medium. After 3 days, differentiation was induced by incubation for two days in the basal neural induction medium ((DMEM/F12 supplemented with N2B27 and NEAA (Life Technologies)) with the addition of nicotinamide (10 mM, Sigma), noggin (50 ng/ml, Peprotech, Rocky Hill, NJ), DKK (10 ng/ml, Peprotech), and IGF1 (10 ng/ml, Peprotech). For the following two days, this differentiation medium was supplemented with FGF2 (5 ng/ml, Peprotech). Subsequently, the cells were cultured in basal neural induction medium supplemented with activin A (100 ng/ml, Peprotech) for two days. For the next seven days, the medium was switched to basal neural induction medium supplemented with activin A (100 ng/ml, Peprotech), CHIR99021 (3 μM, Peprotech), and SU5402 (10 μM, Peprotech). The differentiation process also included a ROCK inhibitor (10 μM of Y-27632 or fasudil). This medium was replaced every day.

Differentiation of NCC was carried out according to previously described procedures with minor modifications [27]. hPSCs were plated at 4 × 10^4^ cells/cm^2^ on plates coated with vitronectin in Essential 6 medium supplemented with CHIR99021 (1.4 μM), FGF2 (100 ng/ml), BMP4 (20 ng/ml), and SB431542 (10 μM, Peprotech). ROCK inhibitors (10 μM of Y-27632 or fasudil) were also present during differentiation. The medium was replaced every day.

After primary differentiation, 13 days for RPE and 7 days for NCC, surviving cells were counted.

### Serum-free 3D PSC aggregation

Aggregated cells were cultured as described previously with minor modifications [12]. Dissociated hPSCs (prepared as above; 1.2 × 10^6^ cells/ml) were seeded onto a StemFIT 3D culture dish (MicroFIT, Republic of Korea) and cultured in DMEM/F12 (Life Technologies) supplemented with 20% Knockout Serum Replacement (KSR, Life Technologies), 2 mM glutamine (Life Technologies), 0.1 mM nonessential amino acids (Life Technologies), 0.1 mM 2-mercaptoethanol (Sigma-Aldrich), 100 U/ml penicillin, and 100 mg/ml streptomycin (GE Life Science, Chicago, IL, USA). 10 μM ROCK inhibitor (Y-27632 or fasudil) was added to the culture medium for 24 hours and cultured for six days in the StemFIT 3D culture dishes. The number and size of aggregates were analyzed using software ImageJ (Wayne Rasband, National Institutes of Health, Bethesda, MD).

### Single-cell colony-forming assay

Accutase (StemCell Technologies) was used to dissociate hPSCs into single cells, which were then plated at 5 × 10^3^ cells/cm^2^ on plates coated with vitronectin in Essential 8 medium. The medium was changed every day and cells were counted after 6 days.

### Statistical analysis

Statistical comparisons between two groups were made using Student’s two-tailed t-test or two-tailed paired t-test. One-way ANOVA with Tukey post hoc analysis using Prism 5 software (GraphPad, San Diego, CA) was used when more than two groups were compared. Data are expressed as mean ± standard deviation (SD).

## Results

### Comparison of fasudil and Y-27632

**Fig 1:**
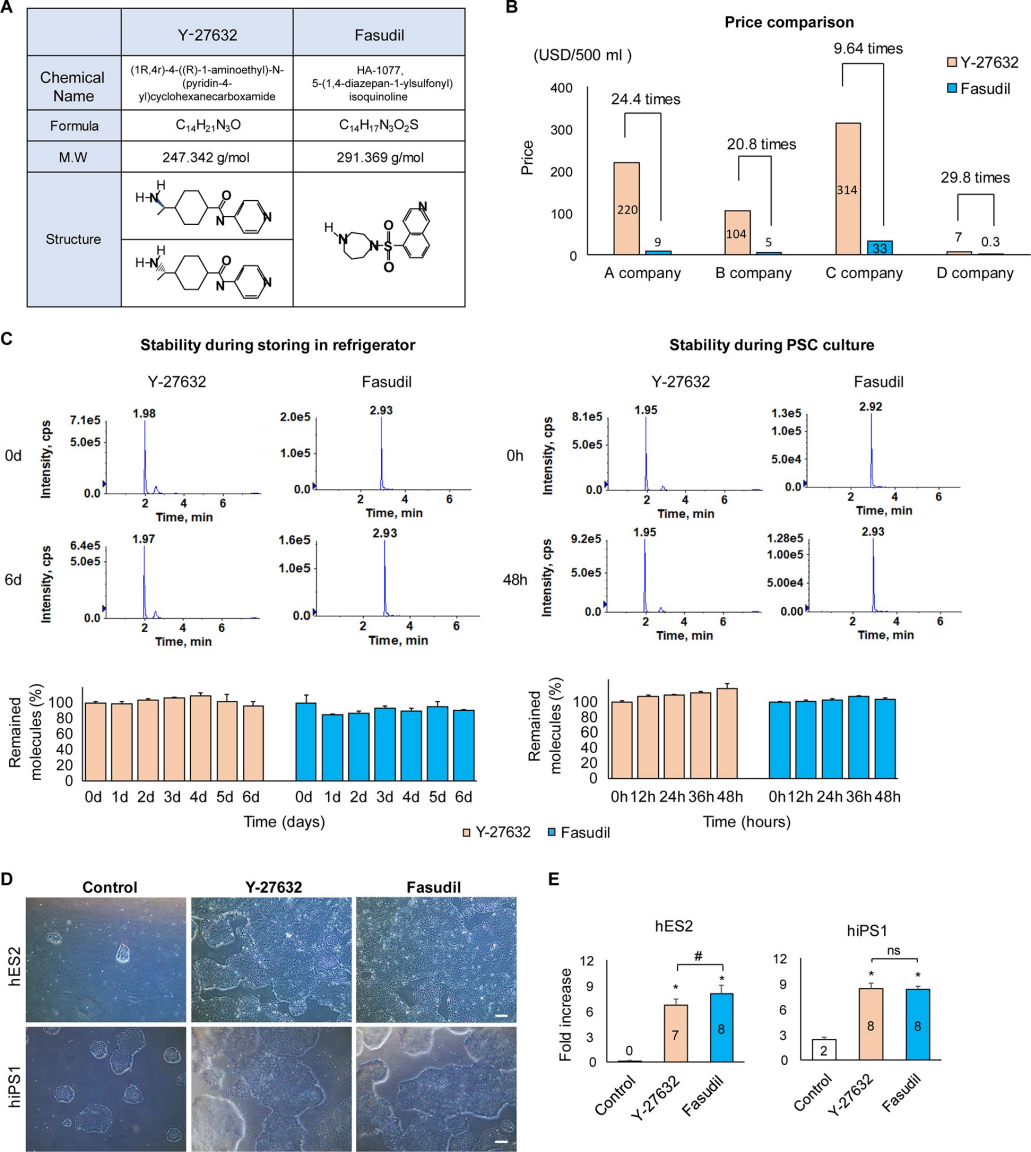
Molecular characteristics of fasudil and Y-27632. **A:** Characteristics of Y-27632 and fasudil. Y-27632 is more difficult to synthesize than fasudil. **B:** Price comparison of Y-27632 and fasudil. Y-27632 is 10 to 30 times more expensive than fasudil, depending on the providing company. **C:** Products ion mass spectrum (top) and stability comparisons (bottom) of fasudil and Y-27632 in media in the refrigerator (2–8°C) or in PSC culture condition (5% CO_2_, 37°C) using LC-ESI/MS/MS. The stability of fasudil was similar to that of Y-27632 (3 biological replicates). **D:** Typical colony morphologies of hPSCs (hES2 and hiPS1) cultured using Y-27632 or fasudil for 7 days after thawing. When treated with fasudil, colonies were larger than the control group. Scale bars = 200 μm. **E:** Fold increases in Y-27632- or fasudil-supplemented cultures for 7 days after thawing. Both fasudil- and Y-27632-treatment stimulated growth (*: *p* < 0.05, 3 biological replicates, 2 technical replicates). The fasudil-treated hES2 group had significantly higher growth than the Y-27632 treated group (#: *p* < 0.05, 3 biological replicates, 2 technical replicates). LC-ESI/MS/MS: liquid chromatography-electrospray ionization tandem mass spectrometry, hPSC: human pluripotent stem cell, hES: human embryonic stem cell, hiPS: human induced pluripotent stem cell.

We wished to develop a method for increasing cell growth during PSC culture and selected fasudil as a candidate small molecule for this purpose. Although fasudil and Y-27632 are both ROCK inhibitors, their molecular structures are remarkably different (Fig 1A) [28]. While Y-27632 is expensive due to its complicated synthesis process, fasudil is relatively simple to synthesize and 10–30 times cheaper than Y-27632 (Fig 1B) [19, 29].

Next, we assessed the stability of fasudil and Y-27632 in a medium for longer times at 2–8°C or 37°C. Both Y-27632 and fasudil were stable for 6 days in the refrigerator (2–8°C) (Fig 1C). They were also stable for up to 2 days in PSC culture (37°C, 5% CO_2_).

Generally, ROCK inhibitors are used to prevent cell death caused by freezing and thawing of hPSCs. To determine whether fasudil has a similar effect as Y-27632, we compared cell numbers after freezing and thawing randomly selected hESC and hiPSC lines. We cultured hPSCs for seven days after freezing and thawing, and measured cell growth (fold increase; cell number after seven days culture/seeded cell number). The hPSCs (hES2 and hiPS1) grew much faster, without morphological changes, when supplemented with Y-27632 or fasudil than the non-treated controls (Fig 1D). Surprisingly, the fasudil-treated in hESCs grew significantly faster (8.1 vs. 6.7, *p* < 0.05) than the Y-27632-treated group (Fig 1E). In the case of the hiPS1, there was no difference between the fasudil- and Y-27632-treated groups (8.4 vs. 8.3). In summary, fasudil not only has a price advantage over Y-27632 but also has a similar or better effect than Y-27632 on growth after thawing of hPSCs.

### Comparable growth of PSCs supplemented with fasudil and Y-27632

**Fig 2:**
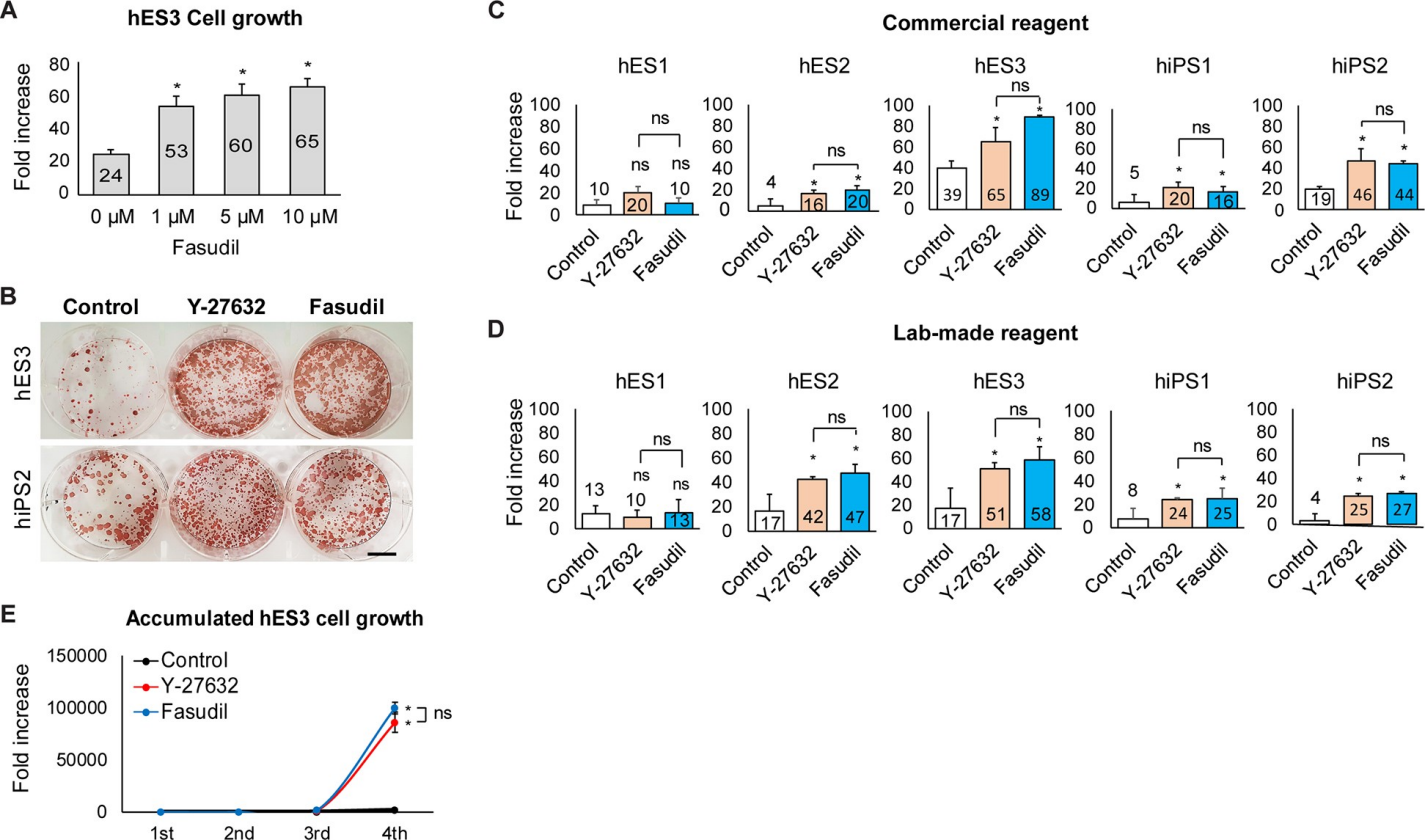
Fasudil enhances the cell growth of hPSCs. **A:** Growth of hES3 cells for 7 days according to fasudil concentration. The growth of hES3 cells at fasudil concentrations of 1, 5, and 10 μM was concentration-independent, and significantly faster than the untreated control (*: *p* < 0.05, 3 biological replicates). **B:** ALP staining of hES3 and hiPS2 cells cultured with Y-27632 or fasudil. Treatment with fasudil or Y-27632 increased the size of PSC colonies. Scale bar = 10 mm. **C, D:** Fold increase of initially seeded hESCs (hES1, 2, and 3) and hiPSCs (hiPS1 and 2) using commercial reagent **C** or lab-made reagent **D.** Growth of all hPSC lines except hES1 increased in response to fasudil or Y-27632 (*: *p* < 0.05, 3 biological replicates). **E:** Increase of hES3 cell numbers with time. After three passages, treatment with fasudil and Y-27632 significantly increased cell numbers compared with the untreated group (*: *p* < 0.05, 3 technical replicates). hPSC: human pluripotent stem cell, hES: human embryonic stem cell, ALP: Alkaline Phosphatase, hiPS: human induced pluripotent stem cell.

To determine the effectiveness of fasudil in PSC culture, we compared the fasudil and Y-27632-mediated hPSC growth of five lines of hPSCs under feeder-free and defined culture conditions. The growth rates of hESCs cultured at 1, 5, and 10 μM were found to be similar, without any morphological changes (Fig 2A). Since the 10 μM ROCK inhibitor-treated samples showed the best growth, we used this concentration in subsequent experiments. ALP staining was carried out on hPSCs (hES3 and hiPS2) cultured with or without ROCK inhibitors to examine their growth. hPSC colony formation was similar for fasudil and Y-27632, while both groups exhibited higher rates of colony formation than the untreated control (Fig 2B).

Next, we compared the fasudil- and Y-27632-mediated growth of five lines of hPSCs under feeder-free and defined culture conditions. Passaging was performed by an enzyme-free method, part of a commercial and laboratory protocol [23]. We evaluated cell growth by the fold increase of cell number. When hPSCs were dissociated and seeded without a ROCK inhibitor, they increased 4 to 39-fold depending on the cell line, whereas, in fasudil, the fold increases were 10 to 89-fold, similar to the Y-27632 culture (*p* < 0.05) (Fig 2C and 2D). The ROCK inhibitors did not affect the growth of hES1.

Furthermore, we measured the number of cells with time. The total number of cells treated with fasudil increased by about 100,000 ± 3,828-fold, which was similar to the group treated with Y-27632 (about 90,000 ± 9,966-fold). However, in the untreated group, the number of cells increased only about 2,400 ± 492-fold (*p* <0.05) (Fig 2E).

**Fig 3:**
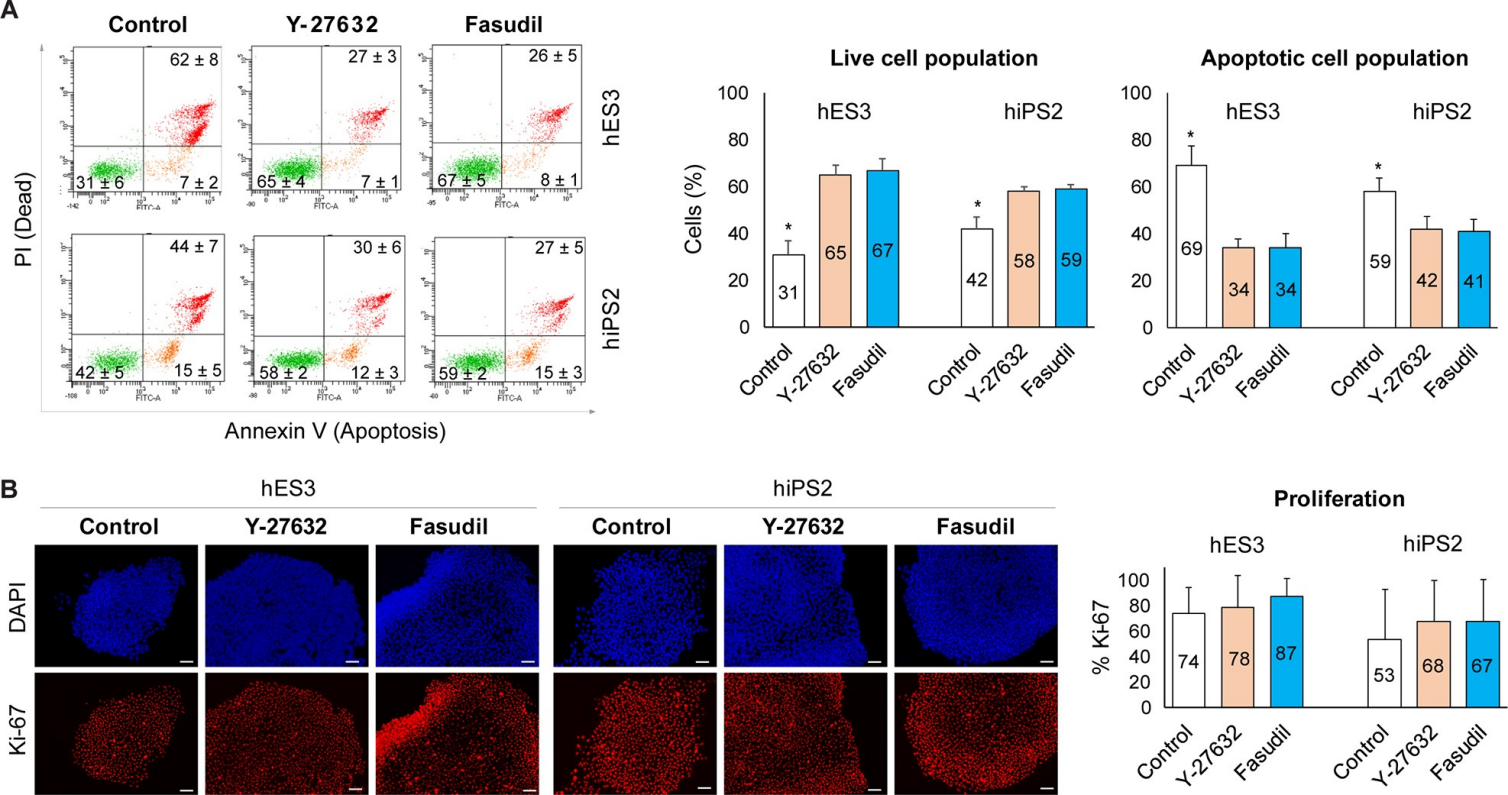
The mechanism of PSC growth. **A:** Analysis of live and apoptotic hES3 and hiPS2 cells (%) 24 hours after seeding at 1 × 10^4^ cells/cm^2^. Compared with the control group, apoptotic cells decreased when treated with fasudil (*: *p* < 0.05; 3 biological replicates). **B:** Cell proliferation measured using immunofluorescence (left) and flow cytometry (right) to examine Ki-67 expression in hES3 and hiPS2 cells 2 days after seeding. There were no significant differences between the groups (3 biological replicates). Scale bar = 100 μm. hPSC: human pluripotent stem cell, hES: human embryonic stem cell, hiPS: human induced pluripotent stem cell.

To understand the mechanisms of PSC growth, we first measured live and apoptotic cell populations using Annexin V and PI. The hES3 and hiPS2 were seeded, then live and apoptotic cell populations were examined 24 hours later (Fig 3A). Both the fasudil- and Y-27632- treated groups had lower apoptotic cell populations than the non-treated controls (34 or 34% vs. 69% for hES3 and 42 or 41% vs. 59% for hiPS2, *p* < 0.05. We also examined Ki-67 protein expression. Since, according to previous reports, the proliferation of hPSCs begins 2 days after passaging [30], hES3 and hiPS2 were cultured for 2 days before the examination. Neither small molecule significantly affected KiPS-67 expression (Fig 3B).

### Maintaining pluripotency following long-term fasudil treatment

**Fig 4:**
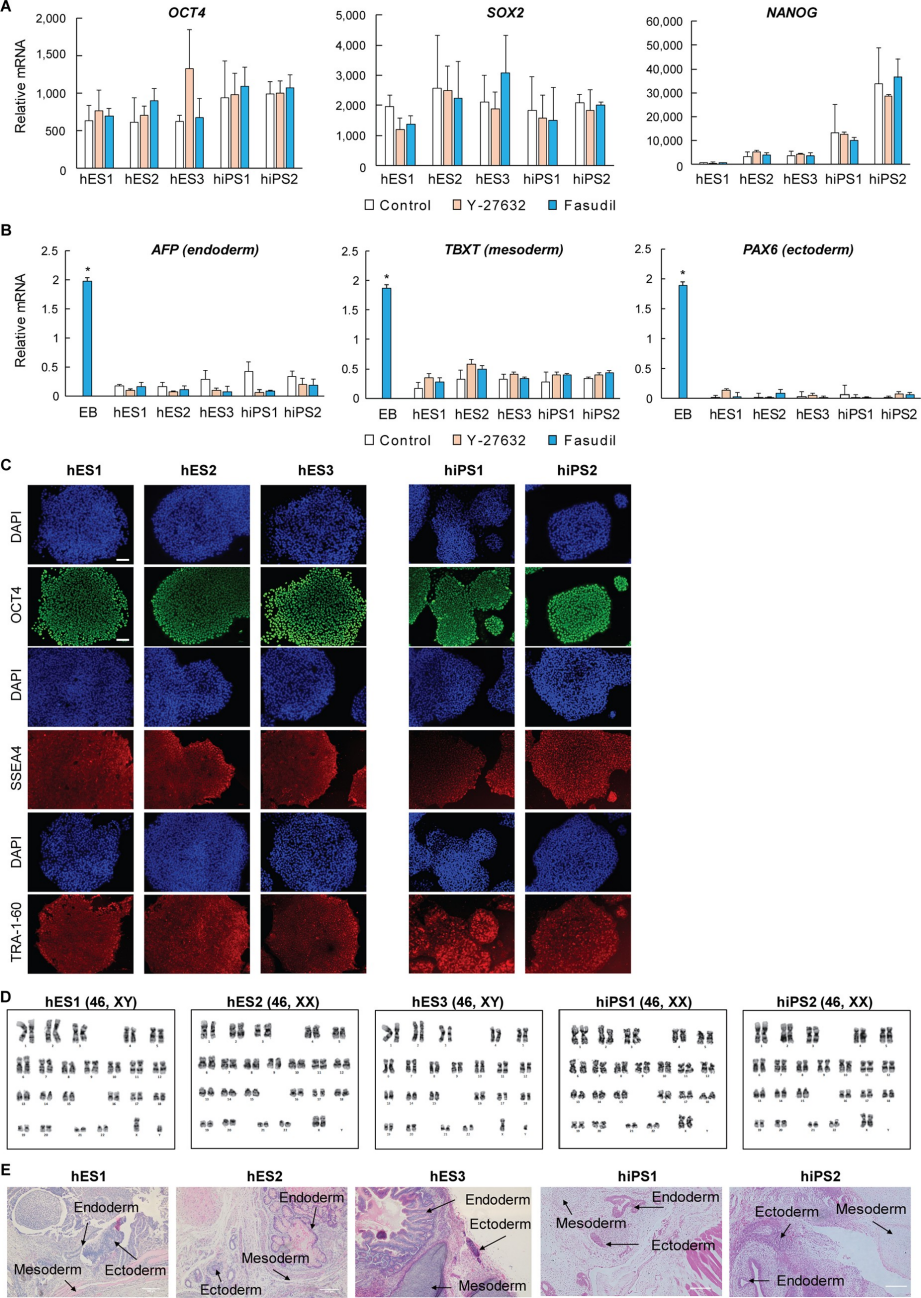
Characterization of hPSCs following fasudil treatment for 2 to 3 months. **A:** Quantitative RT-PCR analysis of *OCT4*, *SOX2*, and *NANOG* expression relative to *GAPDH*. ROCK inhibitors were present only on the first day of passage. The difference between the two treatments was not significant (3 biological replicates, 3 technical replicates). **B**: Quantitative RT-PCR analysis of expression of markers of the 3-germ layer, *AFP*, *TBXT*, and *PAX6*, relative to *GAPDH* (*: *p* < 0.05; versus EBs, 2 biological replicates, 2 technical replicates). EBs were derived from hiPS1 and cultured for 7 days. **C:** Immunofluorescence analysis examining OCT4, SSEA-4, and TRA-1-60 expression, following fasudil treatment for 3 months. All 5 lines of PSC expressed the pluripotency markers. Nuclei were stained with DAPI (blue). Scale bars = 100 μm. **D:** G-banding karyotypes of hPSCs cultured with fasudil for 3 months. None of the five lines of PSC had any abnormality of karyotype. **E:** Ectodermal, mesodermal, and endodermal tissue associated with teratoma formation by five lines of hPSCs after 3 months in fasudil-treated culture. All three germ layers were formed. Scale bars = 200 μm. hPSC: human pluripotent stem cell, EBs: embryoid bodies, hiPS: human induced pluripotent stem cell.

To confirm that fasudil treatment does not affect pluripotency, five hPSC lines were sub-cultured with 10 μM of fasudil over 2 to 3 months and their pluripotency was examined. Representative PSC markers, including *OCT4*, *SOX2*, and *NANOG* were analyzed by qPCR. Individual PSC lines varied but gene expression levels did not differ significantly between the treatment groups (Fig 4A). Next, we examined the gene expression of 3 germ layer markers, *AFP* (endoderm), *TBXT* (mesoderm), and *PAX6* (ectoderm) by qPCR, with hiPS1-derived embryoid bodies (EBs) serving as a positive control. Expression in all 5 lines was significantly lower than in the EBs (*p* < 0.05), and comparable in the control and ROCK inhibitor-treated groups (Fig 4B).

OCT4, SSEA4, and TRA‐1–60 proteins were detected in all the cell lines by immunocytochemistry (ICC) (Fig 4C). Additionally, karyotyping revealed typical chromosome arrangements (Fig 4D). Finally, we injected the five cell lines into immunosuppressed mice (NSG, Jackson Laboratory, Bar Harbor ME) to examine teratoma formation. All the cell lines generated teratomas containing the three germ layers—ectoderm, mesoderm, and endoderm (Fig 4E). These findings demonstrate that fasudil does not affect the pluripotency of PSCs or cause chromosome aberrations and suggest that fasudil is a safe and effective small molecule ROCK inhibitor that can be used in long-term culture of PSCs.

### Effect of fasudil on RPE and NCC differentiation

**Fig 5:**
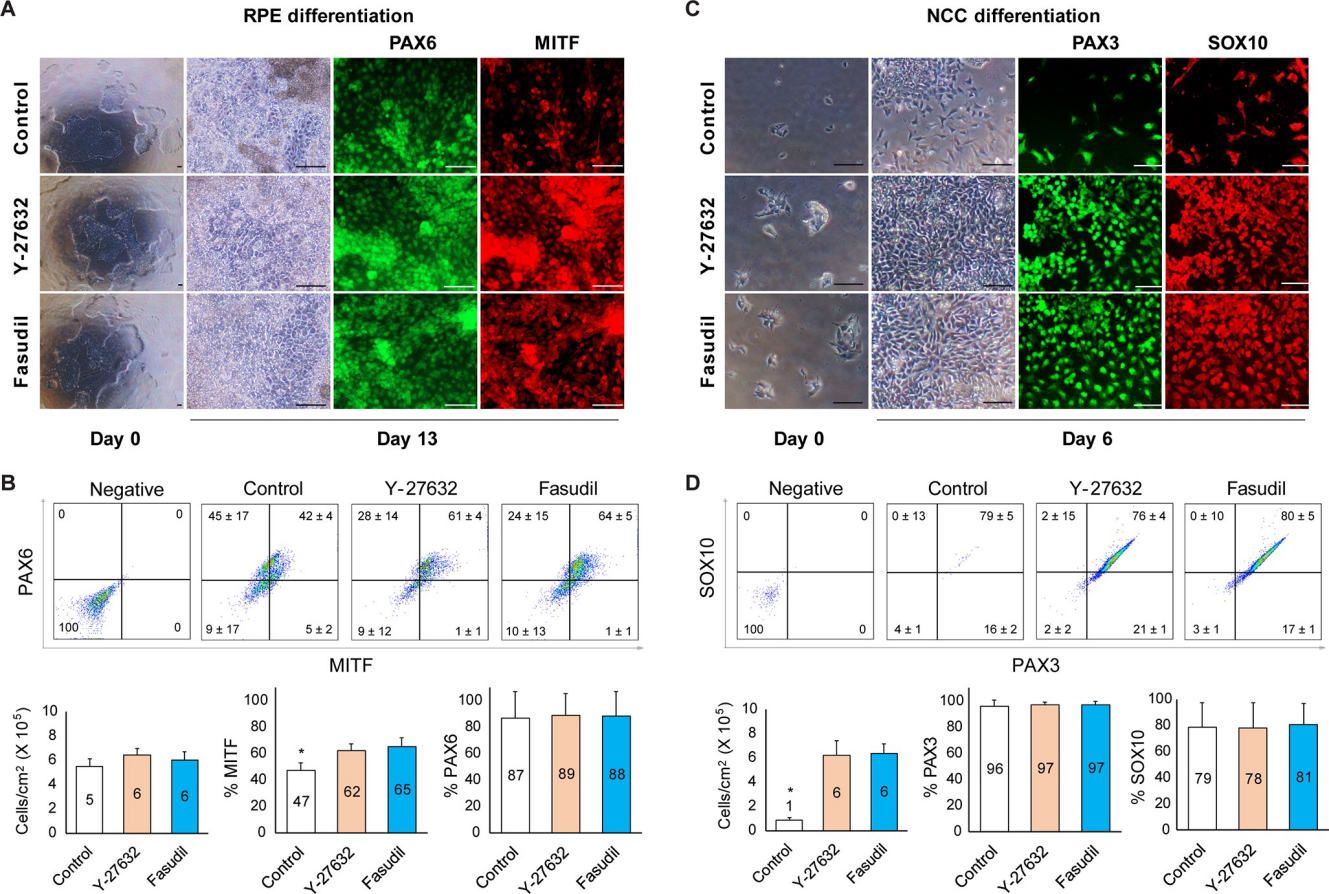
Fasudil promotes differentiation of PSCs. **A:** Expression of RPE-specific markers; PAX6 and MITF examined by immunocytochemistry (ICC) at 13 days. **B:** PAX6 and MITF positive cell populations analyzed by flow cytometry at 13 days. The percentage of PAX6 was similar in the groups, while MITF-positive cells were more frequent in the treated groups than in the untreated control (*: *p* < 0.05, 3 replicates). Fasudil did not affect cell number in RPE differentiation. **C:** The expression of NCC markers; PAX3 and SOX10 by ICC. **D:** PAX3 and SOX10 positive cell population analysis using flow cytometry at 6 days. Fasudil promoted cell survival during NCC differentiation of hiPSCs (*: *p* < 0.05, 3 replicates). RPE: retinal pigment epithelium, NCC: neural crest cell.

To examine the effects of fasudil and Y-27632 on RPE and NCC differentiation by hiPS1 lines, we employed a protocol published previously [26]. After incubation cells treated with fasudil or Y-27632 were in close contact with each other, but cells with the untreated control were distributed relatively loosely (Fig 5A). We examined the expression of the early RPE markers, PAX6 and MITF using ICC, and counted positive cell numbers by flow cytometry. PAX6 was similarly expressed in all three groups, while expression of MITF was significantly higher in the fasudil- and Y-27632-treated groups (62 ± 5% and 65 ± 6%, respectively) than the untreated control (47 ± 6%, *p* < 0.05) (Fig 5B).

Next, we examined NCC differentiation. Differentiated cells in the fasudil-treated and Y-27632-treated cells had grown to cover the plates whereas the cells of the untreated control were present at low confluency (7 ± 3 × 10^4^ cells/cm^2^ vs. 6 ± 1 × 10^5^/cm^2^ for the fasudil-treated group vs the control, *p* < 0.05) (Fig 5C and 5D). The expression of the NCC markers, SOX10, and PAX3, was examined by ICC and counting positive cells using flow cytometry. The differentiation efficiencies of the fasudil- and Y-27632-treated groups were not significantly different from the untreated control group (PAX3: 97% or 96% vs. 96%, SOX10: 81% or 78% vs. 79%), but most of the cells in the control had detached during differentiation (Fig 5D).

### The potential use of Fasudil in a variety of applications

**Fig 6:**
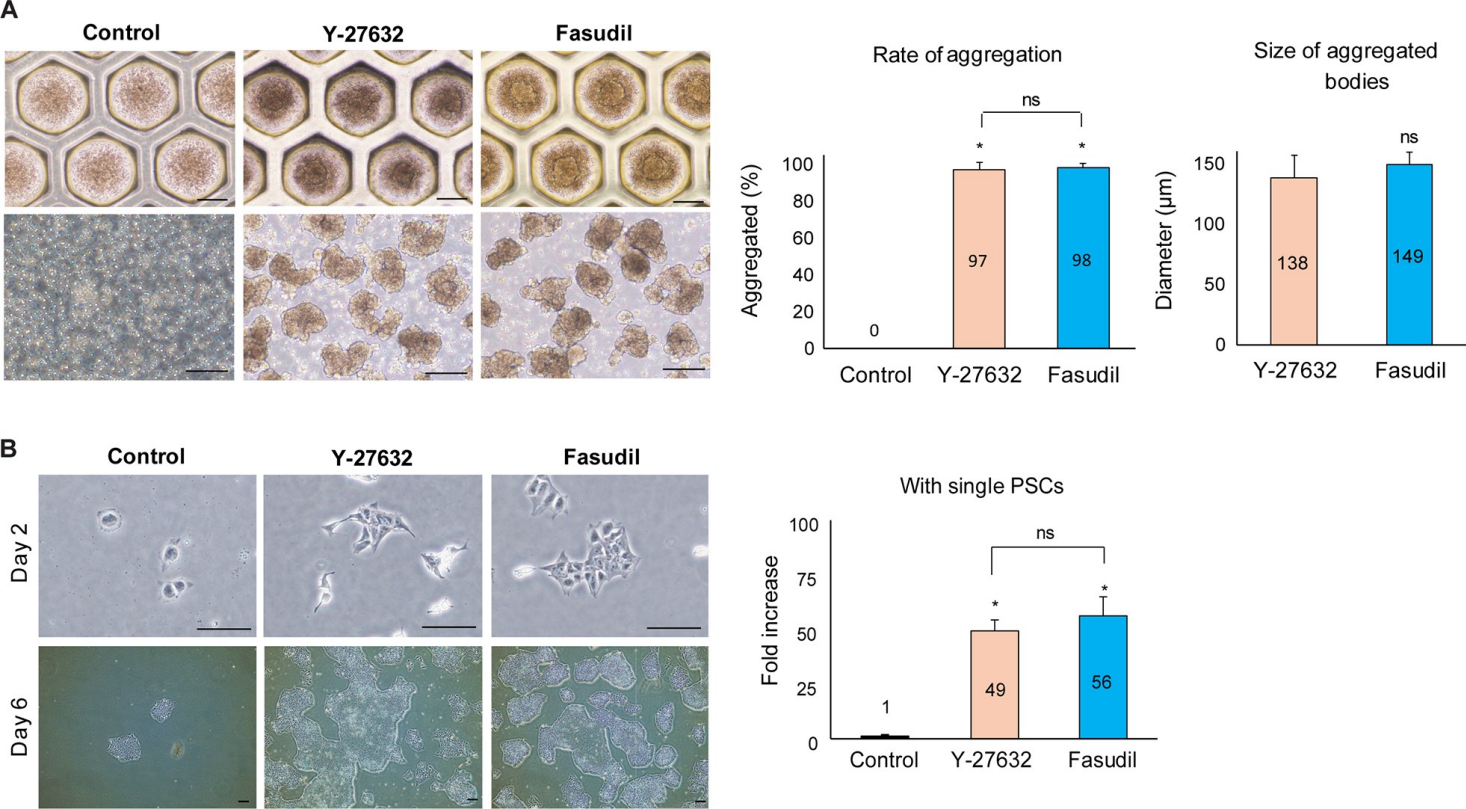
Other applications of fasudil. **A:** Supportive effect of fasudil on hES3 survival and growth in suspension culture. The fasudil-treated groups and Y-27632-treated groups were similar in the rate of formation of cell aggregates and their size; the control cells did not form aggregates. Scale bar = 200 μm (*: *p* < 0.05, 2 biological replicates, 7 technical replicates). **B:** Seven-day cultures of single hES3 cells on vitronectin-coated culture plates in the presence of 10 μM Y-27632 or fasudil. Scale Bar = 200 μm. The growth of hPSCs was faster with Y-27632 or fasudil than in the untreated control (*: *p* < 0.05, 3 technical replicates). hES: human embryonic stem cell, hPSC: human pluripotent stem cell.

To determine whether fasudil might be used in other PSC studies, we investigated the effect of fasudil on 3D aggregation and single-cell culture. To induce the aggregation of hPSCs for 3D culture, dissociated hES3 cells were cultured for six days in suspension (1.2 × 10^6^ cells/ml, 1 ml/35 mm StemFit3D culture dish) using DMEM/F12 medium supplemented with KSR, to which fasudil or Y-27632 was added for the first 24 hours. After 24 hours, the hESCs (hES3) had begun to grow well as floating aggregates, but the control cells remained single (Fig 6A). With fasudil, aggregates formed in almost all the molds (98 ± 2%), and there was no significant difference in comparison with the cells in which Y-27632 was used (97 ± 4%). Also, there was no difference in the size of the aggregates, which were cultured for the aggregate sizes after a total of six days using Y-27632 (138.4 ± 18.6 μm) and fasudil (149.4 ± 10.2 μm).

To confirm the protective effect of fasudil on the growth of single hPSCs, hES3 cells were dissociated into single cells and seeded on a plate with Y27632 or fasudil. Cells were counted after seven days of culture. Treatment with either inhibitor substantially increased cell numbers (56 ± 13 and 49 ± 8 vs. 1 ± 0.6, *p* < 0.05) (Fig 6B).

In summary, fasudil appears suitable for use as a cheaper substitute for Y-27632 in stem cell studies.

## Discussion

ROCK proteins consist of two subunits, ROCK1 and ROCK2, and play crucial roles in numerous cellular functions, including cell contraction, actin organization, cell migration, and proliferation [31]. Cell-cell interactions are essential for colony formation, and study of the roles of the Rho-ROCK-Myosin signaling axis in cell-cell interactions has suggested that signaling mechanisms differ between multipotent and non-pluripotent cell lines [32, 33], and those ROCK inhibitors are useful for culturing human pluripotent stem cells (hPSCs); most researchers use Y-27632 as their preferred ROCK inhibitor.

The ROCK inhibitor fasudil was initially used in the treatment of cancer [34]. Since then, Y-27632, which is more effective in cancer, was developed and used on hPSCs. We were interested in why Y-27632 is the normal choice for PSC studies despite the lower cost of fasudil and its known ability to improve hPSC survival. However, fasudil was not characterized by PSCs through long-term culture.

In an experiment involving ROCK inhibitors, we observed that fasudil promoted hPSC growth. This led to the examination of the action of fasudil on undifferentiated hPSC cultures. We found that when PSCs were frozen and thawed, simple supplementation with fasudil significantly improved survival due to inhibition of the ROCK pathway, which is reported to be one of the most important signaling pathways affecting hPSC survival [35]. Fasudil also improved hPSC growth and the formation of 3D aggregates by preventing apoptosis of dissociated hPSC. The effect of fasudil seems to be due to inhibition of ROCK, which promotes depolymerization of actomyosin, and polymerization of actomyosin is essential for cell survival after detachment [36].

The PSC growth-promoting effects by the ROCK inhibitors varied depending on cell lines. Especially, hES1 did not increase cell growth compared with untreated control. Because the ROCK signal induces cell apoptosis through pMLC and p-cofilin pathway [18], the various expression levels of ROCK, pMLC, and p-cofilin could influence cell growth depending on cell lines [37–39].

We found that inhibition of ROCK by fasudil allowed for long-term culture of hPSCs by maintaining correct gene expression, protein location, function, and karyotype. We also confirmed that the differentiation of hPSC-derived RPE responded to fasudil supplementation. Although these cells were developed for the treatment of age-related macular degeneration (AMD), hPSCs must be cultured for several months to obtain relatively uniform and mature RPEs for transplantation. Fasudil supplementation increased RPE differentiation efficiency. Expression of *MITF* may have been activated by inhibiting the ROCK2 and STAT3 pathways [40, 41]. On the other hand, Fasudil increased the yield of hPSC-derived NCC by inhibiting apoptosis regardless of differentiation efficiency.

## Conclusion

We have shown that fasudil is at least as effective as Y-27632 in stem cell research and may replace Y-27632 in PSC studies. To determine if fasudil could be applied to pluripotent stem cell studies, we used 10 μM fasudil in various experiments such as hPSC culture, differentiation, 3D aggregation, and transport. As a result, fasudil drastically increased the growth of hPSCs. In addition, we confirmed that continuous treatment of fasudil in long term cultures of hPSCs over two months did not affect the expression of pluripotency markers (OCT4, SOX4, NANOG), karyotype and differentiation capacity. Fasudil also increased the differentiation efficiency of RPE and cell survival during NCC differentiation.
